# Parainfluenza virus infections in patients with hematological malignancies or stem cell transplantation: Analysis of clinical characteristics, nosocomial transmission and viral shedding

**DOI:** 10.1371/journal.pone.0271756

**Published:** 2022-07-29

**Authors:** Julia Tabatabai, Paul Schnitzler, Christiane Prifert, Martin Schiller, Benedikt Weissbrich, Marie von Lilienfeld-Toal, Daniel Teschner, Karin Jordan, Carsten Müller-Tidow, Gerlinde Egerer, Nicola Giesen

**Affiliations:** 1 Department of Infectious Diseases, Virology, University Hospital Heidelberg, Heidelberg, Germany; 2 Center for Child and Adolescent Medicine, University Hospital Heidelberg, Heidelberg, Germany; 3 Institute of Virology and Immunobiology, University Hospital Wuerzburg, Wuerzburg, Germany; 4 Department of Internal Medicine V, University Hospital Heidelberg, Heidelberg, Germany; 5 Department of Internal Medicine, HochFranken Hospitals, Munchberg, Germany; 6 Department of Internal Medicine II, University Hospital Jena, Jena, Germany; 7 Leibniz Institute for Natural Product Research and Infection Biology, Hans-Knöll Institut, Jena, Germany; 8 Department of Hematology, Medical Oncology, & Pneumology, University Medical Center of the Johannes Gutenberg University, Mainz, Germany; Taif University, SAUDI ARABIA

## Abstract

To assess morbidity and mortality of parainfluenza virus (PIV) infections in immunocompromised patients, we analysed PIV infections in a hematology and stem cell transplantation (SCT) unit over the course of three years. Isolated PIV strains were characterized by sequence analysis and nosocomial transmission was assessed including phylogenetic analysis of viral strains. 109 cases of PIV infection were identified, 75 in the setting of SCT. PIV type 3 (n = 68) was the most frequent subtype. PIV lower respiratory tract infection (LRTI) was observed in 47 patients (43%) with a mortality of 19%. Severe leukopenia, prior steroid therapy and presence of co-infections were significant risk factors for development of PIV-LRTI in multivariate analysis. Prolonged viral shedding was frequently observed with a median duration of 14 days and up to 79 days, especially in patients after allogeneic SCT and with LRTI. Nosocomial transmission occurred in 47 patients. Phylogenetic analysis of isolated PIV strains and combination with clinical data enabled the identification of seven separate clusters of nosocomial transmission. In conclusion, we observed significant morbidity and mortality of PIV infection in hematology and transplant patients. The clinical impact of co-infections, the possibility of long-term viral shedding and frequent nosocomial transmission should be taken into account when designing infection control strategies.

## Introduction

Respiratory viruses such as influenza, parainfluenza (PIV), respiratory syncytial virus (RSV) and most recently the novel coronavirus SARS-CoV-2 can cause significant morbidity and mortality in immunocompromised patients, in particular in patients with hematologic malignancies or following stem cell transplantation (SCT) [1–3]. While many efforts have been undertaken in research on influenza, much less is known about PIV infections in immunocompromised patients. Several reports describe PIV as a relevant pathogen for immunocompromised patients with mortality rates of PIV-associated lower respiratory tract infection (LRTI) of up to 27% [4–7]. Moreover, PIV is easily transmitted and known to be highly contagious. In contrast to seasonal influenza, PIV infections occur throughout the year. In hematology wards and transplant units, outbreaks of nosocomial PIV infections have been repeatedly reported [6,8–11].

For patients with hematological malignancies presenting with symptoms of respiratory tract infection, testing for respiratory viruses including influenza, PIV and RSV is highly recommended [12]. In contrast to influenza, no specific antiviral therapy has been established against PIV infections [1] and the impact of ribavirin therapy on the outcome of PIV infections remains unclear [13–15].

PIV belongs to the *Paramyxoviridae* family, which comprise enveloped single-stranded negative-sense RNA viruses and is spread by direct contact and aerosols. Based on genetic and antigenic differences PIV types 1–4 have been described, among which PIV type 1 and 3 are classified as members of the genus of *Rubulavirus*, and PIV type 2 and 4 as members of the genus of *Respirovirus* [16–19]. Their major antigenic spike glycolproteins, hemagglutinin neuraminidase and fusion protein, are encoded by HN and F genes, respectively, and are dominant targets for humoral immunity found in all parainfluenza viruses [16]. Further, the HN protein comprises neuraminidase and hemagglutinin functions, and facilitates membrane fusion with host cells by interaction with the F protein [20,21].

Due to its high antigenic and sequence variability the hemagglutinin neuraminidase gene was established as primary target for phylogenetic analysis and typing of PIV [21–25].

Here, we analyze clinical characteristics of PIV infections and risk factors for severe infection in hematological and SCT patients over the course of three years. We assess the extent of nosocomial transmission by combining clinical and molecular data including phylogenetic analysis of viral strains and report on prolonged viral shedding.

## Materials and methods

### Patient population and clinical data assessment

From July 2013 to June 2016, all documented cases of PIV infection in patients with hematologic malignancies or following SCT treated at our institution, a university hospital and transplant center, were included in this analysis. Diagnosis of PIV is established by polymerase chain reaction (PCR) detection of viral RNA in respiratory materials. Patients with PIV infections are regularly re-screened for presence of PIV RNA to determine duration of viral shedding and steer isolation measures.

In this analysis, clinical characteristics and outcome of infected patients were retrospectively evaluated by review of medical charts. PIV-associated LRTI was assumed in case of clinical symptoms of respiratory tract infection (fever, cough, dyspnea) plus atypical pulmonary infiltrates present on thoracic computed tomography (CT) scan in the setting of PIV infection. Severe LRTI was defined as requiring treatment on the intensive care unit (ICU) or fatal outcome. Severe leukopenia was defined as leukocytes < 1000/μl, hypogammaglobulinemia as immunoglobulin G < 6g/l, and prior steroid therapy as prednisolone ≥ 20mg/day or equivalent.

Nosocomial transmission based on clinical data was assumed in patients with detection of PIV infection ≥ 7 days after hospital admission based on the upper limit of the typical incubation period. Assignment to a specific cluster of nosocomial transmission was based on the following epidemiological case definition: identical viral sequence plus overlapping in-patient stay with at least one other cluster patient while both positive for PIV.

Duration of viral shedding was calculated from first to last positive PIV test, patients with only one available positive test were excluded for this analysis.

### PCR and phylogenetic analysis

Viral RNA was extracted from respiratory specimens using the QIAamp® viral RNA mini kit (Qiagen, Hilden, Germany) according to the manufacturer’s protocol. Reverse transcription, amplification and detection of viral RNA was performed with the RealStar® Parainfluenza real-time RT-PCR kit (altona Diagnostics, Hamburg, Germany) on a LightCycler® 480 instrument II (Roche, Mannheim, Germany) according to the manufacturer’s instructions.

Extracted RNA was reverse transcribed using random hexamer primers. Subsequently, PIV HN gene was amplified from cDNA using primers for PIV type 1–4 as previously described or adapted by Villaran et al., Echevarria et al. and Abiko et al. [26–28].

Resulting PCR products with an amplicon length between 430–500 nucleotides were sequenced completely in both directions using Big Dye terminator chemistry version 1.1 on a Prism 3130xl instrument (Applied Biosystems, Darmstadt, Germany). Overlapping sequences were assembled using the SEQMAN II software of the Lasergene package (DNAstar, Madison, USA). Multiple alignments from PIV nucleotide sequences were carried out with the MEGA software version 7 [29]. A phylogenetic tree was generated in MEGA using the maximum-likelihood method and the Tamura-Nei algorithm. Representative reference sequences were obtained from GenBank (http://www.ncbi.nlm.nih.gov) and included in the tree. The statistical significance of the tree topology was assessed by bootstrapping with 1,000 replicates to evaluate confidence estimates. Nucleotide sequences retrieved in this study were deposited in GenBank (accession numbers MT489396-MT489461).

### Statistical analysis

The impact of possible influence factors on morbidity and mortality was analyzed by univariate Chi-square tests. Multivariate logistic regression was performed on a reduced set of variables. Factors that might influence duration of PIV shedding were analyzed by Kruskal-Wallis tests. Multivariate logistic regression was performed regarding the endpoint duration of viral shedding > 14 days. In all analyses, p-values < 0.05 were considered as statistically significant.

This study was approved by the ethics committee of the University of Heidelberg (IRB S-090/2018). Patient records and information were anonymized and de-identified prior to analysis, therefore explicit consent was waived by the ethical committee.

## Results

### Clinical characteristics, morbidity and mortality

**Fig 1:**
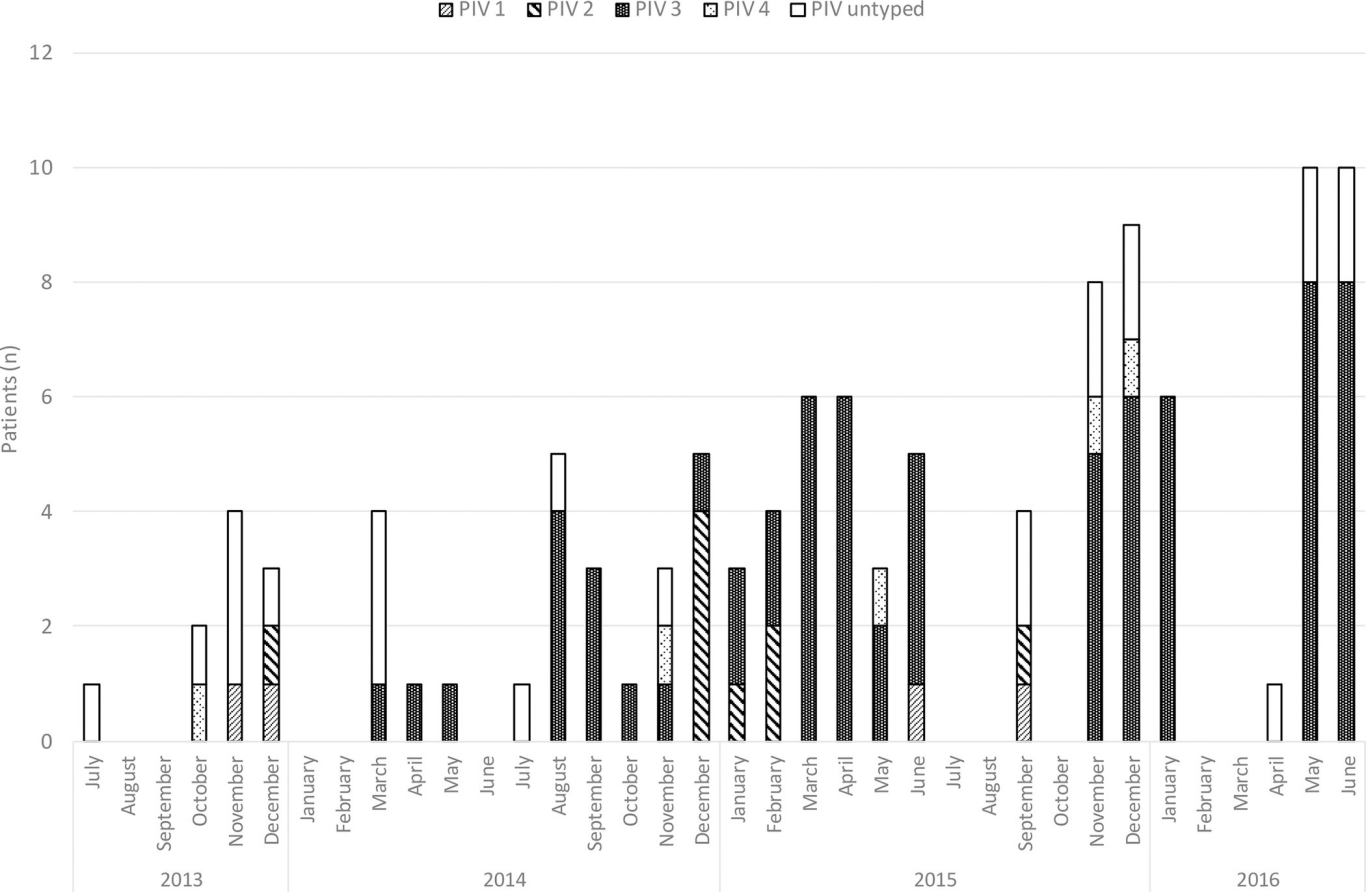
Timeline of parainfluenza virus infections. Untyped PIV: Samples were PCR positive, but could not be sequenced for further typing due to low viral loads.

We identified 109 patients with documented PIV infection between July 2013 and June 2016 (Table 1). The majority of cases was detected during the respective winter and spring seasons (Fig 1). Median age of patients was 60 years [range 26–79], 63% were male. In total 75 patients (69%) had received a SCT (41 allogeneic, 39 autologous, 5 both). Information on PIV subtype was available in 86 cases showing a vast majority of PIV subtype 3 (n = 68; 79%) followed by subtype 2 (n = 9; 10%), 4 (n = 5; 6%), and 1 (n = 4; 5%).

**Table 1:** Clinical characteristics.

	Patients with PIV infections N = 109 (100%)
**PIV type** 1 2 3 4 Data available: n = 78	4 (5) 9 (11) 68 (79) 5 (6)
**Outcome** URTI only LRTI Severe LRTI Fatal outcome	62 (57) 47 (43) 10 (9) 9 (8)
**Age median [range]**	60 years [26–79]
**Male sex**	69 (63)
**Underlying malignancy** Multiple myeloma Lymphoma ALL/LBL AML/MDS other	40 (37) 20 (18) 12 (11) 30 (28) 7 (6)
**Uncontrolled malignancy**	35 (32)
**Stem cell transplant recipient** Allogeneic Autologous PIV infection pre-engraftment	75 (69) 41 (38) 39 (36) 25 (23)
**Graft-versus-host-disease**	21 (19)
**Steroid therapy**	38 (35)
**Severe leukopenia**	50 (46)
**Hypogammaglobulinemia** Data available: n = 85	57 (67)
**Co-infections**	28 (26)
**Nosocomial infection**	47 (43)

Abbreviations: PIV–parainfluenza virus; URTI–upper respiratory tract infection; LRTI–lower respiratory tract infection; ALL–acute lymphoblastic leukemia; LBL–lymphoblastic lymphoma; AML–acute myeloid leukemia; MDS–myelodysplastic syndrome.

Any co-infections were detected in 28 patients (26%), co-infections in respiratory specimens in 11 (10%). Most notable were bacterial co-infections detected in blood cultures (n = 9) or respiratory materials (n = 2), fungal co-infections with aspergillus (n = 3), and co-infections with respiratory viruses (1 FLU-B, 2 RSV, 2 coronavirus).

Regarding outcome, 62 patients (57%) had upper respiratory tract infections (URTI) only, 47 patients (43%) developed a LRTI. A severe LRTI was present in 10 patients. 9/47 patients with LRTI died, resulting in a mortality rate of 19%; 1 patient was put on extracorporeal membrane oxygenation (ECMO) and subsequently recovered. Details on fatal cases are given in Table 2. Within 90 days after PIV infection, 4/62 patients (6%) with PIV-URTI as well as 1 patient with PIV-LRTI who since had recovered from the infection died of unrelated causes.

**Table 2:** Details on cases of fatal parainfluenza virus infection.

#	PIV type	age, years	sex	Underlying malignancy	transplant	Atypical LRTI	Co-infections	Presumed cause of death
1	2	57.1	M	myeloma	auto-allo	yes	*K*. *pneumoniae* (BAL), CMV (BAL), *E*. *coli* (U), *S*. *epidermidis* (BC)	Septic shock, multi-organ failure
2	1	73.1	F	myeloma	-	yes	-	Respiratory failure
3	untyped	69.0	M	PMF	allogeneic	yes	-	ARDS
4	untyped	53.0	M	CLL	allogeneic	yes	-	Respiratory failure
5	3	65.1	F	myeloma	autologous	yes	Aspergillus (BAL)	Respiratory failure
6	3	60.8	F	FL	autologous	yes	Aspergillus (BAL)	Respiratory failure
7	3	50.2	F	AML	allogeneic	yes	-	Cerebral bleeding
8	untyped	78.8	M	DLBCL	-	yes	-	Respiratory failure
9	3	62.9	F	myeloma	autologous	yes	-	Respiratory failure

Abbreviations: PIV–parainfluenza virus; LRTI–lower respiratory tract infection; M–male; F–female; PMF–primary myelofibrosis; CLL–chronic lymphocytic leukemia; FL–follicular lymphoma; AML–acute myeloid leukemia; DLBCL–diffuse large b-cell lymphoma; CMV–cytomegalovirus; BAL–bronchoalveolar lavage; U–urine; BC–blood culture; ARDS–acute respiratory distress syndrome.

### Risk factor analysis regarding morbidity and mortality

Neither type of PIV or underlying hematologic disease had a significant impact on outcome. In particular, no significant association was seen between PIV type 1–4 and development of LRTI (p = 0.81). No increased risk of LRTI, severe LRTI or fatal outcome was seen in patients with prior autologous or allogeneic SCT, even if restricting analysis to patients with SCT within 100 days of PIV diagnosis. Severe leukopenia (p = 0.004), uncontrolled malignancy (p = 0.004), prior steroid therapy (p<0.001), presence of co-infections (p<0.001), and nosocomial transmission (p<0.001) were significantly associated with an increased risk of developing PIV-related LRTI in univariate analysis. In multivariate analysis, severe leukopenia (p = 0.01), prior steroid therapy (p = 0.001), and presence of co-infections (p = 0.01) remained significant risk factors for development of LRTI (Table 3).

**Table 3:** Multivariate risk factor analysis regarding development of LRTI.

Factor	p-value	HR	95% CI
**Allogeneic SCT**	0.89	0.92	0.29;2.95
**Autologous SCT ≤ 100 days**	0.42	0.58	0.15;2.22
**Steroid therapy**	0.001	6.03	2.15;16.95
**Severe leukopenia**	0.01	4.96	1.46;16.90
**Age ≥ 65 years**	0.39	1.67	0.52;5.37
**Co-infections**	0.01	4.04	1.32;12.36

Abbreviations: LRTI–lower respiratory tract infection; HR–hazard ratio; 95% CI– 95% confidence interval; SCT–stem cell transplantation.

With respect to fatal outcome, presence of respiratory tract co-infections (p = 0.02) and prior steroid therapy (p<0.001) showed a significant impact (p = 0.001) in univariate analysis, a trend was seen for male sex (p = 0.05). No parameters reached statistical significance in multivariate analysis.

Patients with PIV infection pre-engraftment did not show a significantly prolonged time-to-engraftment compared to patients with infection post-engraftment neither in case of allogeneic not autologous transplantation (p = 0.81 and p = 0.63, resp.).

Regarding antiviral therapy, ribavirin is not standard of care for PIV infection at our institution. In this cohort, only one patient with PIV LRTI received ribavirin and survived, making any conclusions as towards its effectiveness speculative.

### Viral shedding

**Fig 2:**
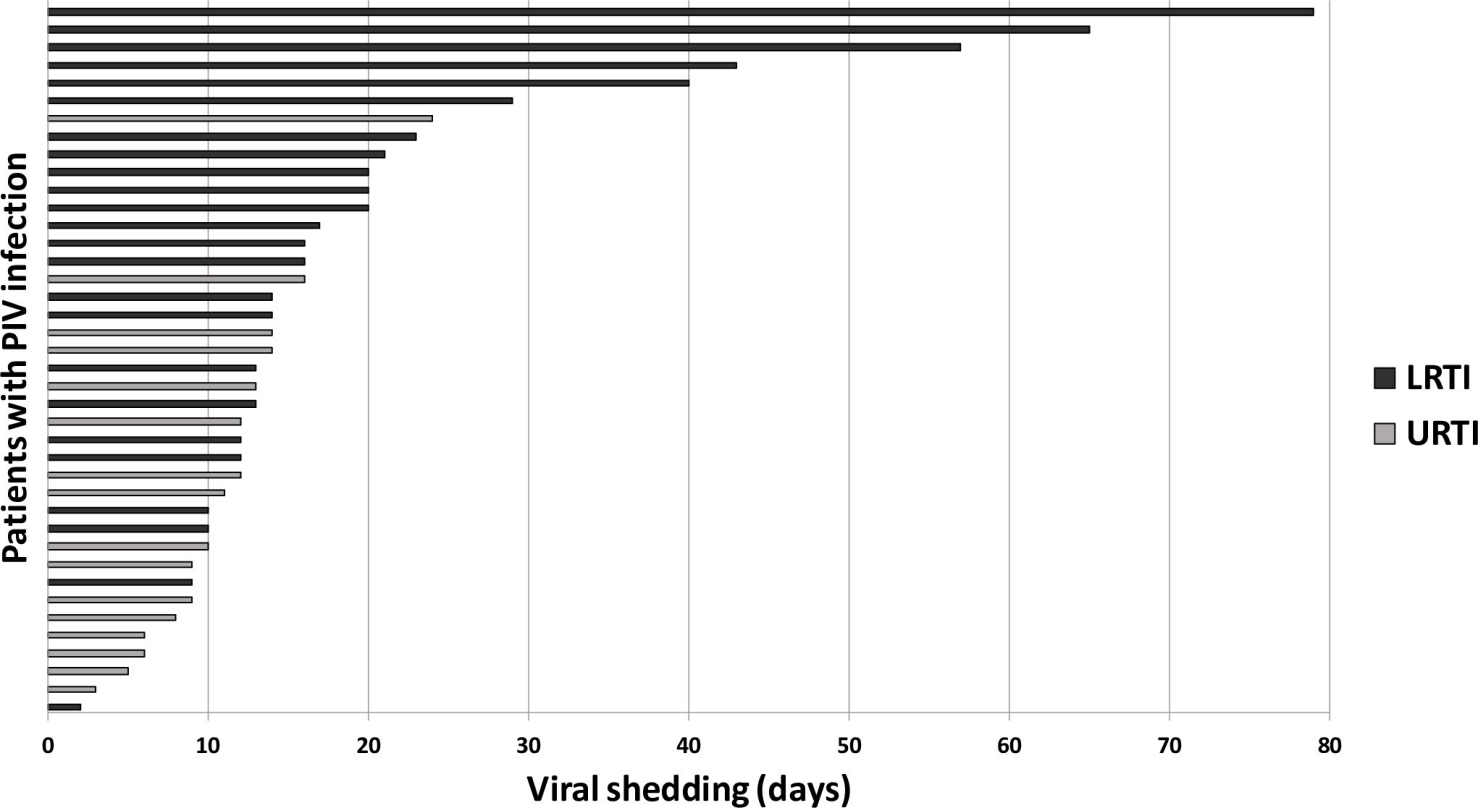
Duration of viral shedding in patients with PIV infection. Data on viral shedding was available in 40 patients with consecutive tests for PIV. Patients with URTI and LRTI are designated by green and red bars, resp.

Data on viral shedding was available in 40 patients. Median duration of viral shedding was 14 days (range 3–79 days, Fig 2). In univariate analysis, male sex (p = 0.02), severe leukopenia (p = 0.01), prior steroid therapy (p = 0.03), nosocomial acquisition (p = 0.005), LRTI (p = 0.001) and presence of co-infections (p = 0.04) were significantly more frequently associated with prolonged viral shedding. In multivariate analysis, a trend was seen for prolonged viral shedding in patients with allogeneic transplantation (p = 0.07), presence of LRTI (p = 0.09), and severe leukopenia (p = 0.09) (Table 4). Interestingly, available data from 2 patients who acquired PIV infection prior to engraftment after allogeneic SCT showed remarkably prolonged viral shedding for 57 and 79 days, respectively.

**Table 4:** Multivariate risk factor analysis regarding prolonged viral shedding > 14 days.

Factor	p-value	HR	95% CI
**Allogeneic SCT**	0.07	8.63	0.84;88.72
**Steroid therapy**	0.61	1.62	0.26;10.12
**LRTI**	0.09	6.29	0.76;52.21
**Severe leukopenia**	0.09	7.42	0.73;74.90

Abbreviations: HR–hazard ratio; 95% CI– 95% confidence interval; SCT–stem cell transplantation; LRTI–lower respiratory tract infection.

### Phylogenetic analysis and assessment of nosocomial transmission

**Fig 3:**
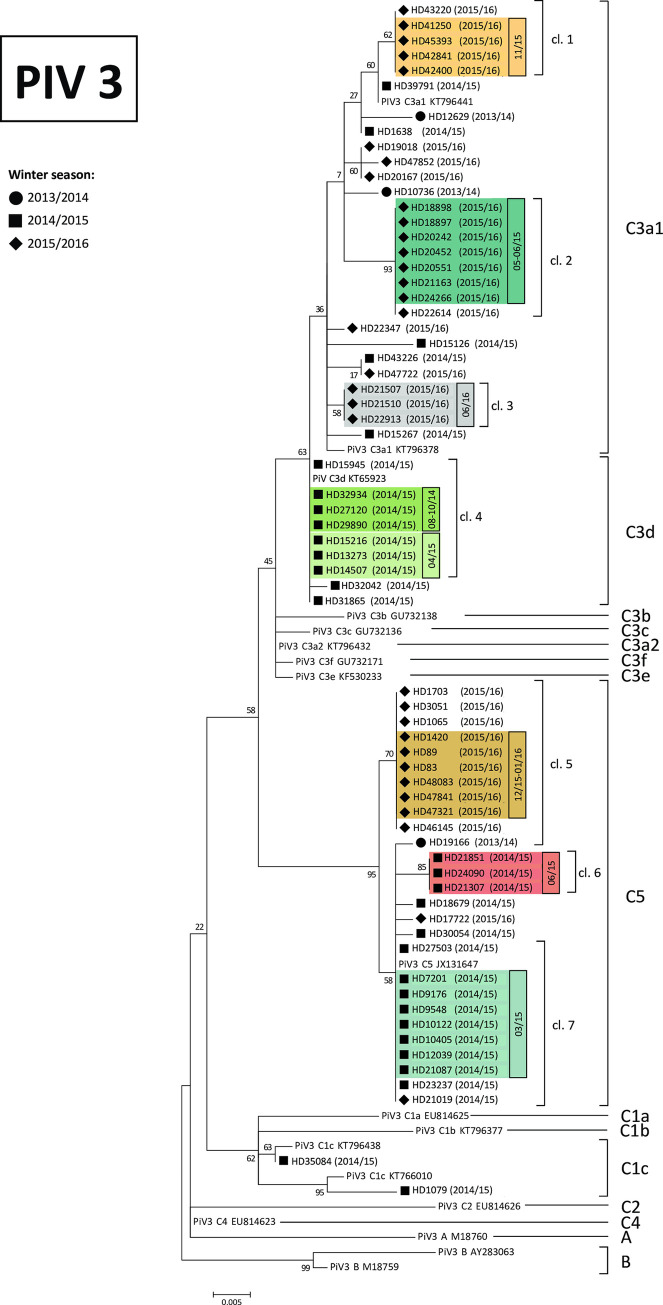
Phylogenetic analysis of PIV strains including information on clusters of nosocomial transmission. Phylogenetic tree for nucleotide sequences of PIV-3 strains were constructed with maximum-likelihood method with 1,000 bootstrap replicates using MEGA 7 software. Heidelberg strains are named with their strain identifier followed by the winter season of isolation in brackets. Reference strains representing known genotypes were retrieved from GenBank and included in the tree (labels include genotype followed by accession number). The genotype assignment is also shown on the right by brackets. Bootstrap values greater than 70% are indicated at the branch nodes. Clinically suspected nosocomial infections matching identical sequence clusters (cl. 1–7) are highlighted in color (one cluster of suspected nosocomial infection in the outpatient setting is highlighted in grey), time of infection is shown in black circled box on the right. The scale bar represents the number of nucleotide substitutions per site. cl. = cluster.

Nosocomial transmission based on clinical data was apparent in 47 patients (43%). Of these, genetic identification of the PIV strain was possible in 38 patients. Combining information on nosocomial transmission according to clinical definition with phylogenetic data on viral strains, we could identify seven clusters of nosocomial PIV infections consisting each of patients with clinically defined nosocomial PIV infection, overlapping stays as in-patients and identical viral sequence. The identified clusters included up to seven patients each and were spread over a period of 23 months (Fig 3). Two nosocomial clusters of three patients each were located within the same phylogenetic cluster but occurred during different time periods (PIV3 C3d, 08-10/14, 04/15). Out of 38 patients with nosocomially acquired PIV infection and available sequence data, 33 patients (87%) could be assigned to one of the clusters. In addition, seven patients with presumably community-acquired PIV infection showed viral sequences identical to one of the clusters, three of these were hospitalized within the PIV incubation period but shorter than the upper limit of standard incubation period and might be in fact nosocomial cases. Furthermore, three patients with community-acquired PIV infection formed an additional cluster (PIV3 C3a1, 06/2016). All three were treated during the presumed time of infection in the allogeneic transplant outpatient clinic, thus nosocomial transmission in the waiting area might be conceivable.

## Discussion

This multi-season study of PIV infections in a diverse population of patients with hematologic malignancies including both SCT and non-SCT patients shows significant morbidity and mortality with nearly half of infected patients developing pneumonia and a subsequent LRTI-associated mortality rate of 19%. The incidence of severe courses of PIV infection seen here is within range of those reported by others, taking into consideration that most published studies focused on high-risk populations such as patients with leukemia or following SCT [5,7,14,30,31]. In our study population, SCT status was not a significant risk factor for severe outcome. This highlights the role of PIV as an important pathogen in patients with hematologic malignancies both within and outside the SCT setting.

While PIV type 3 has been associated with an increased incidence of LRTI in hematologic patients [32] we could not detect a significant association between PIV type and development of LRTI. However, in our cohort PIV type 3 was responsible for nearly 4 in 5 of overall PIV infections. Of interest, among the six fatal cases with information on PIV type, two were associated with PIV other than type 3, namely type 1 and 2, respectively.

Prior steroid therapy, severe leukopenia and presence of co-infections were identified as significant risk factors for PIV-LRTI. We observed bacterial, fungal and viral co-infections. Of interest, in five cases co-infections with other respiratory viruses including two cases of co-infection with coronavirus (non-COVID-19) were detected. However, there was no noticeable associated increase in morbidity in these cases. Presence of co-infections has been repeatedly described as a risk factor for severe PIV infection [14,30,33]. Recently, invasive pulmonary aspergillosis (IPA) as a complication of severe influenza has been gaining a lot of attention with reported incidence rates of 30% of immunocompromised ICU patients and high associated mortality [34]. We observed three cases of IPA and PIV co-infection. All three required treatment on the ICU, two subsequently died, one patient recovered following ECMO therapy. This demonstrates the potential severity of IPA in immunocompromised patients with PIV infection. It is therefore important to aim for thorough microbiological work-up in patients with PIV infection, particularly in the immunocompromised host, in order to detect possible co-infections and adapt antimicrobial therapy accordingly.

Therapeutic options targeting PIV are currently very limited. Antiviral therapy with ribavirin is highly controversial with most studies failing to show a significant impact on LRTI development or mortality [15]. Intravenous immunoglobulin administration may be considered as supportive therapy [1]. An antiviral agent currently in phase III development for PIV infection is the sialidase fusion protein fludase (DAS181). First data suggest fludase may be an effective treatment strategy for PIV LRTI in immunocompromised patients [35]. However, until effective antiviral agents are broadly available, infection control measures remain the cornerstone against PIV infections.

To optimize infection control measures, assessment of viral shedding can be a helpful strategy. We could demonstrate prolonged viral shedding of up to 79 days, particularly in patients with LRTI, severe leukopenia, and allogeneic SCT recipients. While too few to gain statistical significance, PIV infection pre-engraftment of allogeneic SCT seemed a high-risk constellation for prolonged viral shedding. Long-term viral shedding of influenza, PIV, and RSV in immunocompromised patients has been previously reported by our group with especially long periods of nearly a year observed for RSV [36] and has also been described for the novel coronavirus SARS-CoV-2 [37]. The possibility of long-term viral shedding has to be kept in mind when devising infection control strategies as it might facilitate nosocomial transmission and outbreaks.

Clinically suspected nosocomial transmission supported by sequence analysis was a frequent finding in our study cohort despite comprehensive hygienic measures implemented at our institution. This highlights the high contagiousness of PIV, especially in such a vulnerable patient population. Outbreaks of PIV on hematology and oncology wards and in SCT units have been repeatedly reported including both outbreaks of a single and multiple virus strains [6,8,10,11,38,39]. We here describe multiple clusters of nosocomial transmission in immunocompromised patients outside of a traditional outbreak setting covering a long time period. The combination of clinical and phylogenetic data allowed a detailed case-by-case analysis and to illustrate the route and extent of nosocomial transmissions. Clusters of nosocomial transmission could be observed during all four seasons reflecting the presence of PIV throughout the year, highlighting the need to implement adequate infection control measures at any time. Circle threshold values in real time PCR as proxy for viral load did not show any association with LRTI nor severe LRTI in our cohort. However, it is very conceivable that a prolonged period of viral shedding, such as here observed in allogeneic transplant patients increases the risk of nosocomial transmission. At our institution, isolation of not only infected patients but also their contact patients for the length of the possible incubation period is standard of care which might have contributed to stop the development of larger outbreaks despite the obviously repeated introduction of PIV into this highly vulnerable patient population. Barrier methods addressing the entire population at risk such as a universal mask strategy if in contact with SCT patients have also been shown to be effective in reducing PIV infections [40].

As a retrospective analysis, this study has several limitations. Detailed documentation of clinical symptoms as well as stringent follow-up swabs to determine duration of viral shedding were not available in all patients, especially in the out-patient setting. Furthermore, testing for PIV was limited to patients. Thus, no information on PIV infections among health-care workers or patients’ relatives was available which would have added useful aspects with regard to chains of transmission.

In conclusion, we could demonstrate significant morbidity and mortality of PIV infections in a diverse population of hematologic and SCT patients. Nosocomial transmission occurred frequently and might be facilitated by long-term viral shedding in immunocompromised patients highlighting the need for comprehensive infection control management. Further prospective studies are necessary to design optimal strategies with regard to infection prevention and transmission control in this vulnerable patient population, and to further develop efficient vaccination and treatment options.

## References

[1] von Lilienfeld-Toal M, Berger A, Christopeit M, Hentrich M, Heussel CP, Kalkreuth J, et al. Community acquired respiratory virus infections in cancer patients-Guideline on diagnosis and management by the Infectious Diseases Working Party of the German Society for haematology and Medical Oncology. Eur J Cancer. 2016; 67: 200–212. doi: 10.1016/j.ejca.2016.08.015 27681877 PMC7125955

[2] Chemaly RF, Ghosh S, Bodey GP, Rohatgi N, Safdar A, Keating MJ et al. Respiratory viral infections in adults with hematologic malignancies and human stem cell transplantation recipients: a retrospective study at a major cancer center. Medicine (Baltimore). 2006; 85(5): 278–87. 16974212 10.1097/01.md.0000232560.22098.4e

[3] He W, Chen L, Chen L, Yuan G, Fang Y, Chen W, et al. COVID-19 in persons with haematological cancers. Leukemia. 2020; 34(6): 1637–1645. doi: 10.1038/s41375-020-0836-7 32332856 PMC7180672

[4] Falsey AR. Current management of parainfluenza pneumonitis in immunocompromised patients: a review. Infect Drug Resist. 2012; 5: 121–7. doi: 10.2147/IDR.S25874 22893749 PMC3418768

[5] Marcolini JA, Malik S, Suki D, Whimbey E, and Bodey GP. Respiratory disease due to parainfluenza virus in adult leukemia patients. Eur J Clin Microbiol Infect Dis. 2003; 22(2): 79–84. doi: 10.1007/s10096-002-0864-4 12627280

[6] Maziarz RT, Sridharan P, Slater S, Meyers G, Post M, Erdman DD, et al. Control of an outbreak of human parainfluenza virus 3 in hematopoietic stem cell transplant recipients. Biol Blood Marrow Transplant. 2010; 16(2): 192–8. doi: 10.1016/j.bbmt.2009.09.014 19781656 PMC7172256

[7] Nichols WG, Corey L, Gooley T, Davis C, and Boeckh M. Parainfluenza virus infections after hematopoietic stem cell transplantation: risk factors, response to antiviral therapy, and effect on transplant outcome. Blood. 2001; 98(3): 573–8. doi: 10.1182/blood.v98.3.573 11468152

[8] Lee AV, Bibby DF, Oakervee H, Rohatiner A, Ushiro-Lumb I, Clark DA et al. Nosocomial transmission of parainfluenza 3 virus in hematological patients characterized by molecular epidemiology. Transpl Infect Dis. 2011; 13(4): 433–7. doi: 10.1111/j.1399-3062.2011.00603.x 21466639

[9] Jalal H, Bibby DF, Bennett J, Sampson RE, Brink NS, MacKinnon S, et al. Molecular investigations of an outbreak of parainfluenza virus type 3 and respiratory syncytial virus infections in a hematology unit. J Clin Microbiol. 2007; 45(6): 1690–6. doi: 10.1128/JCM.01912-06 17392447 PMC1933051

[10] Piralla A, Percivalle E, Di Cesare-Merlone A, Locatelli F, and Gerna G. Multicluster nosocomial outbreak of parainfluenza virus type 3 infection in a pediatric oncohematology unit: a phylogenetic study. Haematologica. 2009; 94(6): 833–9. doi: 10.3324/haematol.2008.003319 19377073 PMC2688575

[11] Harvala H, Gaunt E, McIntyre C, Roddie H, Labonte S, Curran E, et al. Epidemiology and clinical characteristics of parainfluenza virus 3 outbreak in a Haemato-oncology unit. J Infect. 2012; 65(3): 246–54. doi: 10.1016/j.jinf.2012.04.011 22546619

[12] Hirsch HH, Martino R, Ward KN, Boeckh M, Einsele H, and Ljungman P. Fourth European Conference on Infections in Leukaemia (ECIL-4): guidelines for diagnosis and treatment of human respiratory syncytial virus, parainfluenza virus, metapneumovirus, rhinovirus, and coronavirus. Clin Infect Dis. 2013; 56(2): 258–66. doi: 10.1093/cid/cis844 23024295 PMC3526251

[13] Pinana JL, Hernandez-Boluda JC, Calabuig M, Ballester I, Marin M, Madrid S, et al. A risk-adapted approach to treating respiratory syncytial virus and human parainfluenza virus in allogeneic stem cell transplantation recipients with oral ribavirin therapy: A pilot study. Transpl Infect Dis. 2017; 19(4). doi: 10.1111/tid.12729 28544152

[14] Chemaly RF, Hanmod SS, Rathod DB, Ghantoji SS, Jiang Y, Doshi A, et al. The characteristics and outcomes of parainfluenza virus infections in 200 patients with leukemia or recipients of hematopoietic stem cell transplantation. Blood. 2012; 119(12): 2738–45; quiz 2969. doi: 10.1182/blood-2011-08-371112 22246027

[15] Shah DP, Shah PK, Azzi JM, and Chemaly RF. Parainfluenza virus infections in hematopoietic cell transplant recipients and hematologic malignancy patients: A systematic review. Cancer Lett. 2016; 370(2): 358–64. doi: 10.1016/j.canlet.2015.11.014 26582658 PMC4684719

[16] Henrickson KJ. Parainfluenza viruses. Clin Microbiol Rev; 2003; 16(2):242–64. doi: 10.1128/CMR.16.2.242-264.2003 12692097 PMC153148

[17] Almajhdi FN. Hemagglutinin-neuraminidase gene sequence-based reclassification of human parainfluenza virus 3 variants. Intervirol; 2015; 58(1):35–40. doi: 10.1159/000369208 25592955

[18] Kuhn JH, Becker S, Ebihara H, Geisbert TW, Johnson KM, Kawaoka Y, et al. Proposal for a revised taxonomy of the family Filoviridae: classification, names of taxa and viruses, and virus abbreviations. Arch Virol; 2010; 155(12):2083–103. doi: 10.1007/s00705-010-0814-x 21046175 PMC3074192

[19] Vainionpää R, Hyypiä T. Biology of parainfluenza viruses. Clin Microbiol Rev; 1994; 7(2):265–75. doi: 10.1128/CMR.7.2.265 8055470 PMC358320

[20] Tsurudome M, Ohtsuka J, Ito M, Nishio M, Nosaka T. The Hemagglutinin-Neuraminidase (HN) Head Domain and the Fusion (F) Protein Stalk Domain of the Parainfluenza Viruses Affect the Specificity of the HN-F Interaction. Front Microbiol; 2018;9:391. doi: 10.3389/fmicb.2018.00391 29593671 PMC5859044

[21] Mao N, Ji Y, Xie Z, Wang H, Wang H, An J, et al. Human parainfluenza virus-associated respiratory tract infection among children and genetic analysis of HPIV-3 strains in Beijing, China. PLoS ONE;2012;7(8):e43893. doi: 10.1371/journal.pone.0043893 22937119 PMC3429441

[22] Kawano M, Bando H, Yuasa T, Kondo K, Tsurudome M, Komada H, et al. Sequence determination of the hemagglutinin-neuraminidase (HN) gene of human parainfluenza type 2 virus and the construction of a phylogenetic tree for HN proteins of all the paramyxoviruses that are infectious to humans. Virol; 1990; 174(1):308–13. doi: 10.1016/0042-6822(90)90081-2 2152995

[23] Košutić-Gulija T, Slović A, Ljubin-Sternak S, Mlinaric-Galinovic G, Forcic D. Genetic analysis of human parainfluenza virus type 3 obtained in Croatia, 2011–2015. J Med Microbiol; 2017;66(4):502–10. doi: 10.1099/jmm.0.000459 28463659

[24] Henrickson KJ, Savatski LL. Genetic variation and evolution of human parainfluenza virus type 1 hemagglutinin neuraminidase: analysis of 12 clinical isolates. J Infect Dis; 1992; 166(5):995–1005. doi: 10.1093/infdis/166.5.995 1328413

[25] Elusah J, Bulimo WD, Opanda SM, Symekher SL, Wamunyokoli F. Genetic diversity and evolutionary analysis of human respirovirus type 3 strains isolated in Kenya using complete hemagglutinin-neuraminidase (HN) gene. PLoS ONE;2020; 15(3):e0229355. doi: 10.1371/journal.pone.0229355 32155160 PMC7064169

[26] Villaran MV, Garcia J, Gomez J, Arango AE, Gonzales M, Chicaiza W, et al. Human parainfluenza virus in patients with influenza-like illness from Central and South America during 2006–2010. Influenza Other Respir Viruses. 2014; 8(2): 217–27. doi: 10.1111/irv.12211 24286248 PMC4186470

[27] Echevarria JE, Erdman DD, Swierkosz EM, Holloway BP, and Anderson LJ. Simultaneous detection and identification of human parainfluenza viruses 1, 2, and 3 from clinical samples by multiplex PCR. J Clin Microbiol. 1998; 36(5): 1388–91. doi: 10.1128/JCM.36.5.1388-1391.1998 9574711 PMC104834

[28] Abiko C, Mizuta K, Aoki Y, Ikeda T, Itagaki T, Noda M, et al. An outbreak of parainfluenza virus type 4 infections among children with acute respiratory infections during the 2011–2012 winter season in Yamagata, Japan. Jpn J Infect Dis. 2013; 66(1): 76–8. doi: 10.7883/yoken.66.76 23429092

[29] Kumar S, Stecher G and Tamura K. MEGA7: Molecular Evolutionary Genetics Analysis Version 7.0 for Bigger Datasets. Mol Biol Evol. 2016; 33(7): 1870–4. doi: 10.1093/molbev/msw054 27004904 PMC8210823

[30] Seo S, Xie H, Leisenring WM, Kuypers JM, Sahoo FT, Goyal S, et al. Risk Factors for Parainfluenza Virus Lower Respiratory Tract Disease after Hematopoietic Cell Transplantation. Biol Blood Marrow Transplant. 2019; 25(1): 163–171. doi: 10.1016/j.bbmt.2018.08.021 30149147 PMC6310631

[31] Seo S, Xie H, Campbell AP, Kuypers JM, Leisenring WM, Englund JA, et al. Parainfluenza virus lower respiratory tract disease after hematopoietic cell transplant: viral detection in the lung predicts outcome. Clin Infect Dis. 2014; 58(10): 1357–68. doi: 10.1093/cid/ciu134 24599766 PMC4001290

[32] Lefeuvre C, Salmona M, Bondeelle L, Houdouin V, Feghoul L, Jacquier H, et al. Frequent lower respiratory tract disease in hematological patients with parainfluenza virus type 3 infection. J Med Virol; 2021; 93(11):6371–6. doi: 10.1002/jmv.27243 34324206

[33] Russell E, Yang A, Tardrew S, and Ison MG. Parainfluenza Virus in Hospitalized Adults: A 7-Year Retrospective Study. Clin Infect Dis. 2019; 68(2): 298–305. doi: 10.1093/cid/ciy451 29961826

[34] Schauwvlieghe A, Rijnders BJA, Philips N, Verwijs R, Vanderbeke L, Van Tienen C, et al. Invasive aspergillosis in patients admitted to the intensive care unit with severe influenza: a retrospective cohort study. Lancet Respir Med. 2018; 6(10): 782–792. doi: 10.1016/S2213-2600(18)30274-1 30076119

[35] Salvatore M, Satlin MJ, Jacobs SE, Jenkins SG, Schuetz AN, Moss RB, et al. DAS181 for Treatment of Parainfluenza Virus Infections in Hematopoietic Stem Cell Transplant Recipients at a Single Center. Biol Blood Marrow Transplant. 2016; 22(5): 965–70. doi: 10.1016/j.bbmt.2016.02.011 26904972

[36] Lehners N, Tabatabai J, Prifert C, Wedde M, Puthenparambil J, Weissbrich B, et al. Long-Term Shedding of Influenza Virus, Parainfluenza Virus, Respiratory Syncytial Virus and Nosocomial Epidemiology in Patients with Hematological Disorders. PLoS One. 2016; 11(2): e0148258. doi: 10.1371/journal.pone.0148258 26866481 PMC4750950

[37] Aydillo T, Gonzalez-Reiche AS, Aslam S, van de Guchte A, Khan Z, Obla A, et al. Shedding of Viable SARS-CoV-2 after Immunosuppressive Therapy for Cancer. N Engl J Med. 2020; 383(26): 2586–2588. doi: 10.1056/NEJMc2031670 33259154 PMC7722690

[38] Berrueco R, Anton A, Rives S, Catala A, Toll T, Ruiz A, et al. Multiplex real-time PCR for prompt diagnosis of an outbreak of human parainfluenza 3 virus in children with acute leukemia. Infection. 2013; 41(6): 1171–5. doi: 10.1007/s15010-013-0498-8 23821486 PMC7100800

[39] Kothari A, Burgess MJ, Crescencio JCR, Kennedy JL, Denson JL, Schwalm KC, et al. The role of next generation sequencing in infection prevention in human parainfluenza virus 3 infections in immunocompromised patients. J Clin Virol. 2017; 92: 53–55. doi: 10.1016/j.jcv.2017.05.010 28531552 PMC5521260

[40] Sung AD, Sung JAM, Thomas S, Hyslop T, Gasparetto C, Long G, et al. Universal Mask Usage for Reduction of Respiratory Viral Infections After Stem Cell Transplant: A Prospective Trial. Clin Infect Dis. 2016; 63(8): 999–1006. doi: 10.1093/cid/ciw451 27481873 PMC5036914

