# Supplementary material for: Parainfluenza virus infections in patients with hematological malignancies or stem cell transplantation: Analysis of clinical characteristics, nosocomial transmission and viral shedding
Source: PLoS One. 2022 Jul 29;17(7):e0271756. doi: 10.1371/journal.pone.0271756 (PMC9337657; doi:10.1371/journal.pone.0271756)
Supplement: Attachment — Submitted filename: Comment.DOCX [file pone.0271756.s001.DOCX]

To authors,

This is an interesting and well written manuscript that throws light on an important virus that caused outbreaks and high mortality among the immunocompromised patients.

My recommendations

Please include the epidemiological case definition used to assign patients to an outbreak or cluster. Please include any detected risk factors that increased transmission, and if these risk factors were addressed.
